# ‘…What God and the Angels Know of us?’ Character, Autonomy, and best Interests in Minimally Conscious State

**DOI:** 10.1093/medlaw/fwx051

**Published:** 2017-11-13

**Authors:** Giles Birchley

**Affiliations:** Centre for Ethics in Medicine, Bristol Medical School, Population Health Sciences, University of Bristol, UK

**Keywords:** Minimally conscious state, best interests, character, narrative

## Abstract

Determining the best interests of incapacitated patients has been observed to be an opaque area of the law, and this is no less so in decisions about the (non-)treatment of patients in the minimally conscious state. A systematic examination of the way best interests are used in judgments relating to this population suggests that narratives involving the character of the patient frequently form an important plank of judicial reasoning. Since insights into the concept of best interests may be gained by an engagement with the philosophy of well-being, I identify the court’s character-based approach with perfectionist theories of well-being. These use human nature to furnish an objective list of abilities needed for human flourishing. Guided by the Mental Capacity Act (MCA), this list becomes focused primarily on autonomy. Incapacitated patients are assumed to have wishes, but to lack agency. Judges search for these wishes in narratives about the patient and supply the means to exercise these wishes. This analysis suggests three concerns about the court’s approach: first, by placing so great a weight on autonomy, the law offers an impoverished account of human nature; secondly, adversarial law encourages partial determinations of character, and this raises concerns about whether the courts are equipped to explore the complexities of character narratives; and, thirdly, experimental psychology indicates character is not as predictable as an assessment under MCA requires. While character narratives may unburden decision-makers, this analysis suggests the limits of autonomy may have been exceeded in this area of the law.

## I. INTRODUCTION

*‘There is … a very great difference between a man's reputation and a man's character, for reputation is what men think and say of us, while character is what God and the angels know of us.*’^1^

Price Collier’s 1892 sermon draws on a familiar distinction between the person known to others and the unmediated person known only to the Almighty. This distinction applies not just to theology. It draws on questions familiar to social science^2^ and psychology^3^ about how much a person can be known to others, how much is known only to the self, and how much can never be known. Our answers determine, *inter alia*, the limits of our ability to respect autonomy when we make decisions for others.

Using analysis of cases about (non)treatment in minimally conscious state (MCS), this article explores this issue. In particular I consider the way character (which I broadly construe as personal qualities, personality, and past and present behaviours) is used in judgments. While the way character evaluation operates in criminal law has been explored,^4^ this article focuses upon civil law. In particular I explore the way that character is used to inform determinations of best interests. I observe that assessment of character is being used to play a (sometimes large) evidential role in sketching the probable motivations of patients in MCS. I suggest that character is used in judgments to supply a narrative from which to adjudge the patient’s wishes and thereby determine their best interests. This approach is led by the autonomy-enhancing spirit of the Mental Capacity Act 2005 (MCA 2005), section 4(6). This states that, absent an advance decision to refuse treatment (ADRT) or lasting power of attorney (LPA),^5^ decisions made in the best interests of an incapacitated person must give due consideration to the wishes and feelings, beliefs, and values that would influence the person’s decision.

My exploration of this state of affairs is two-pronged. I seek to clarify the way character is used to determine best interests by undertaking a philosophical analysis. Once this analysis is developed I use these insights to develop a critique of current practice. We can gain philosophical insight into the best interests standard by drawing on theories of prudential value, otherwise known as the philosophy of well-being.^6^ By examining these theories, I identify connections between best interests judgments and so-called ‘perfectionist theories of well-being’ (hereafter: *perfectionist approaches*).^7^ These suggest that personal well-being is achieved through the expression of particular aspects of human nature.^8^ By comparing the theoretical processes by which perfectionist approaches are derived with the practice of the law, I raise three key criticisms about the ways character informs the judgment of best interests in MCS: first, by seeking narratives that privilege autonomy, the law overlooks the diversity of possible approaches to frailty and dying held by patients. Secondly, adversarial law encourages partial determinations of character in MCS, raising concerns about the suitability of the courts to explore the complexities of character. Thirdly, experimental psychology indicates character is not as predictable as an assessment under MCA 2005 section 4(6) requires, raising questions about the ability of character to inform determinations of specific wishes.

My overall intention is to critically inform the development of the best interests test. An ideal best interests test must take account of the (formally stated) prior wishes of the patient, the deleterious effects of their disease (including apparent pain and distress), and the views of those closest to the patient including the expert views of both family and healthcare professionals (without under- or over-stating the value of each). Such a process requires evaluations of science and ethics, which are transparent and open about the limitations of each discipline. Many of these elements are present in current case law. However, I argue that while analysis of character provides a sometimes narratologically satisfying background to decisions, it should not suffice for an account of prior wishes where these are not present. Nor, in the case of a severe neurological impairment, should past character be treated as a gloss to current ambiguous behaviours.

## II. BACKGROUND

### A. Best Interests in MCS

MCS is a type of disorder of consciousness where patients show consistent and reproducible signs of awareness, albeit at a very limited level.^9^ MCS has been recognised in medical guidelines since 2002. Decisions about non-treatment of patients in both MCS and permanent vegetative state (PVS, a related disorder where the patient shows no consistent signs of awareness) must come before the courts.^10^ For some time following *Bland*,^11^ where the courts first considered non-treatment of PVS patients, MCS was not distinguished from PVS. Records show a number of cases where patients showed signs of minimal awareness.^12^ In these early cases, while medical witnesses were divided over whether PVS could be diagnosed, the courts were minded to allow withdrawal on the basis set out in *Bland*.^13^ Central to this basis was the suggestion in *Bland* that a patient in PVS has no prospect of recovery. As a result, treatment of a PVS patient is futile and best interests need no further consideration. Since *W v M*,^14^ the approach to MCS has differed from PVS. *W v M* established that treatment of patients in MCS may not be futile. Instead, best interests should be determined on a case-by-case basis on the aggregate balance of interests. Subsequently, in *James*,^15^ futility was judged by the Supreme Court to rest only on whether the patient would have thought the treatment was futile, rather than the treatment’s prospect of cure. Consideration of best interests in MCS may therefore lead to treatment (eg *W v M*) or may not (eg *Briggs*^16^).

*Bland* ^17^ also confirmed the possibility of the patient’s advance decision determining their best interests once incapacitated. Subsequently a 20-year-old verbal request for non-treatment of a patient in a terminal state of reduced consciousness,^18^ was considered too remote and too little informed of the fatal consequences to constitute a valid ADRT. The approach of the courts is now led by the MCA 2005. This indicates that the patient’s autonomous wishes guide decision-making when they have the capacity to decide. Where the patient lacks capacity, decisions will either: follow the patient’s prior wishes in the form of an advance decision; be guided by the individual(s) named by the patient in a LPA for health and welfare, or; assess the patient’s best interests by ascertaining wishes and feelings, beliefs, and values and any other information that they would consider relevant to the decision. The MCA 2005 placed rigorous requirements on ADRTs with fatal consequences. These must be in writing, signed, and witnessed if they are to be applicable.^19^ Because in common law, best interests is held to encompass a full range of considerations including ‘medical, emotional and all other issues’,^20^ informal statements about the values, wishes, and beliefs of patients have a role in determining best interests. However, the courts have consistently distinguished the best interests test from ‘substituted judgment’.^21^ This is a doctrine in US law that allows the judge, informed by the incapacitated patient’s past remarks and attitudes, to make the decision on behalf of the incapacitated patient that she considers the patient would prefer if they momentarily regained capacity. The best interests test is nevertheless held to contain a strong element of substituted judgment.^22^ The increasing influence of informal reports of prior wishes in determining the best interests of patients in MCS,^23^ has led some commentators to suggest personal autonomy is on the march.^24^

### B. Best Interests in Philosophy

The best interests test has long been argued to allow subjective value judgments.^25^ Some commentators argue that this allows decision-makers an excessive discretion.^26^ Others contend that this lack of prescriptivity allows decision-makers to sensitively respond to unique aspects of cases.^27^ Some clarity about the types of values, and coherence of approaches, used in the best interests test can be gained by considering philosophical perspectives. Philosophers identify the best interests test with prudential value theory, the study of personal well-being.^28^ Also known as the philosophy of well-being, this theory contains three overarching accounts.^29^*Desire* accounts suggest that our lives go best if we are able to satisfy our wants or preferences. What is most desired by the individual is what is good for that individual, and the thwarting of a desire is equally harmful to that individual’s well-being. The argument that our well-being is enhanced by the fulfilment of our desires has a strong pedigree in western philosophy. Desire-based accounts are at the centre of economic and political theory.^30^ In its purest form, a desire account may suggest the well-being of patients in MCS is judged on the basis of their antecedent wishes, and a patient who expressed a prior wish for (non-)treatment should be respected. Objections to desire theories include the concern that fulfilment of uninformed, capricious, or fleeting desires seem unlikely to enhance well-being.

*Hedonistic* accounts argue that the best lives are those in which pleasure or happiness is maximised, while pain is the source of harm to well-being.^31^ If both wanted and unwanted pleasures are taken to be equal, the idea that pleasure is the source of well-being may be a compelling way of assessing the well-being of patients in MCS who seem happy despite their disability. Such an account might favour non-treatment of an MCS patient only if the patient is in pain, or alternatively, suggest non-treatment where incapacity is so great that no pleasure can be experienced. Objections to hedonistic theories suffer objections that unpleasant experiences can be experienced positively (I may be glad to feel exhaustion after a visit to the gym as it shows it has done me good).

*Objective list* accounts contend that lives go best according to fixed criteria. These accounts suggest that a universal list of things that are good for people can be identified. Our well-being is greater if we have more of the things on this list, and less if we have fewer.^32^ There are a great many objective list theories, and content varies, but objective lists commonly exclude direct appeals to desires or pleasures (we need not desire or enjoy what is good for us) and include a plurality of goods (there are a number of things that are good for us).^33^ Proponents claim that objective list theories are the theories of well-being most likely to succeed. They claim objective lists are intuitively more plausible than other theories. Further, because they can exclude things which seem obviously harmful to well-being, they allow objective lists to nullify the strongest objections to desire and hedonistic theories. One exemplary objective list theory contains the following items: moral goodness, rational activity, development of abilities, having children and being a good parent, knowledge and awareness of beauty.^34^ Since the lives of patients in MCS are unlikely to contain many of these items, their levels of well-being might be judged to be low, and therefore non-treatment might be justified, even if the patient has expressed no wish for this and seems happy. However, critics argue that objective lists tend to lack any satisfying theoretical basis for including the items they do, suggesting they are capricious and the conclusions they guide potentially unfounded.^35^

Anticipating that one or more of these accounts will, in some form, be present in judgments of best interests in MCS, we can surmise that an investigation into the theoretical basis of these judgments will yield a richer and more informative understanding of the best interests test.^36^

### C. BABEL: Best Interests in Medical Ethics and Law

This article arises from BABEL, a Wellcome Trust funded seed project based on the research question ‘How are the best interests of incapacitated patients interpreted and applied in judicial decision-making?’ The project aims to build research communities that explore how bioethics and law interact in the interpretation and application of the best interests test. While the ultimate intention is to consider best interests in a wide frame, the first phase of the project has explicitly focused on small areas of judgments, including where the non-treatment of patients in MCS is in question. This provided an achievable focus for activities in the first year, as well as identifying methodologies and concepts for more sustained investigation as the project matures.

The methods by which this research was undertaken are described in detail elsewhere^37^; however, in brief, a search was conducted for all judgments until July 2016 that discussed non-treatment of patients either in a MCS, or experiencing a disorder of consciousness that appeared (to the author) diagnostically similar to MCS (ie where patients were judged to be in a vegetative state despite questions being raised about their diagnosis). The resulting case series comprised seventeen cases.^38^ The judgments were imported into *NVivo 10* software and analysed using a thematic approach.^39^ This allowed the close scrutiny of the facts, approaches, and values^40^ that occur within this body of case law, and the identification of broad themes that could be comprehensively illustrated using extracts from the judgments.

### D. Themes Arising from the Cases

The cases reveal a complex and changing approach to MCS. The themes that inform these approaches could be framed in a variety of ways. For example, parallel cases starkly highlight changes of approach: *W v KH*^41^ and *M v N*,^42^ both concern patients with multiple sclerosis (MS) at the end of their lives whose families believe would not wish to be treated. Such cases were used to consider the predictability of legal approaches.^43^ The cases allowed examination of the type and weight of values that are used in each case.

Here I concentrate on a third theme, the role of character. Detailed studies of patient character are immediately apparent in the judgments of Hayden J (eg *M v N*^44^ and *Sheffield v TH*^45^). Examination of other cases revealed this approach occurred in sixteen of the seventeen cases in our series, and encompassed the character of the family or other witnesses as well as the patient.^46^ While we might expect criminal law to take an interest in the characterological aspects of witnesses and defendants, this finding was somewhat unexpected where the best interests of incapacitated patients were considered. Since the theme was so abundant, and patient character was the most interesting aspect of the use of character, this article concentrates exclusively on the way the character of the patient is portrayed. As my discussion will indicate, character is a contested concept; nevertheless, a broad definition is sufficient for the purposes of this article. I take character to encompass the qualities of an individual drawn from their past and current behaviours. In the cases in the series, these qualities are used to furnish a narrative of what makes that individual who they are. From the narrative is abstracted likely motivations and intentions.

The law is no stranger to evaluating character. Criminal law tends to focus on intention rather than motive, and the *mens rea* has sometimes been observed to contain a characterological dimension.^47^ For example, feminist legal scholars have analysed the role of gendered assumptions about character in the construction of verdicts in criminal trials. Nicholson compares two criminal cases where battered women had killed their abusive partners in similar circumstances.^48^ He argues the courts employed biographical factors unrelated to the case to place the women on a ‘spectrum of femininity’.^49^ These evaluations place each woman on a continuum of fickleness or fidelity, submissiveness or assertiveness, caringness or selfishness, and are observed to colour the facts and lead to opposite outcomes. Such characterisation may not be inevitable. A strictly consequentialist account of the law could conceivably eschew judgments of character and rely only on the evidence of the harmful outcome (ie causation), on the basis that punishing those responsible is most effective in controlling crime.^50^ Yet, arguably, widespread public respect is essential for a functional system of law. This creates a strong incentive for the law to be seen to punish wrongdoers according to dessert. Character also plays its part in civil law. This may occur in the use of paradigmatic example against which the behaviour of parties is compared, for example, Munby P’s discussion of good parenting in the child custody case of *H-B.*^51^ Judgments of the character of witnesses may also be made, and considered indicative of the (un)reliability of their testimony. This is can be seen where similar family behaviours are characterised as in dramatically different ways. Thus, the resistance to withholding treatment by a family in *St George’s v P*^52^ is portrayed as steadfast and measured, while the opposition of a family to withholding treatment in *County Durham v PP*^53^ is vacillating and bellicose. Finally, in the absence of a valid advance decision or LPA, the Court of Protection may explore the character of the incapacitated patient to assay their wishes and values in accordance with the MCA 2005, section 4(6). In these cases, character informs a narrative that ultimately underwrites a best interests judgment.

I note, prior to our discussion, that I may be accused of taking some terminological liberties. Some consider ‘personality’ to denote amoral traits like introversion, while ‘character’ denotes moral traits, such as honesty.^54^ Whether character in this moral sense can be empirically investigated is debated in psychology.^55^ Since any distinction does not affect my arguments, unless making specific reference to personality psychology, I use the term ‘character’ hereafter.^56^

## III. PATIENT CHARACTER IN MCS

Not all cases in the series employ patient character in a sophisticated way.^57^ Its absence may emphasise other features of the patient, for example, their extreme disability. I lack the space here for analysis of character or its absence in every case. To economise, I will discuss four exemplar cases, beginning with *W v M*.^58^ These exemplars were selected both to illustrate the range of ways that character can play a part in judgments, and demonstrate that it is a commonplace approach to judgecraft.

### *A. W v M:* Playful and Flirtatious

*W v M,* ^59^ the first case in which MCS is explicitly distinguished by the courts,^60^ contains two competing portrayals of the patient. Her family characterises M as thwarted by her disability while M’s carers sketch her rich range of emotional and interpersonal behaviours.

M had sustained severe brain damage following an acute infection, and spent 7 years presumed to be in PVS. An application was made to the court by the family for withdrawal of Artificial Nutrition and Hydration (ANH). Expert investigations undertaken for the purposes of the application concluded that M was in MCS, rather than PVS. Her family gave evidence that M’s antecedent behaviour would nevertheless support withdrawal of treatment. The care home staff offer a different portrait that emphasises M’s quality of life, including her response to music and to her carers. The court accepted that there had been no improvement in M’s condition for some years, and that the chances of further recovery were remote. Nevertheless Baker J concluded that M’s level of consciousness prevented withdrawal of ANH. He refused the application, finding that M’s quality of life might be improved with stimulation, and that respect for the sanctity of life should prevail.

At a fundamental level *W v M* hinges on a contested view of who the patient is, and therefore, what constituted her character. Is M the independent person who would have rejected a life of dependence? Or is she a disabled person whose life retains meaning and pleasure, given the right stimuli?^61^ This is far more than a test of prior versus present wishes, but an investigation into identity. Great efforts are made to portray M’s character in particular ways.^62^

M’s family describes M as active, strong willed, and caring, attributes she manifestly does not have the ability to express in her current state. For example, her partner said M was a:

strong-willed person who was not one to shy away from things she believed in … she was someone who was pretty focused on what she wanted out of life - someone who knew her own mind.^63^

For her sister, M’s MCS was a fundamental impediment to M’s self-expression: ‘She can't enjoy things like she used to do, how can being taken out change her condition?’^64^

The testimony of M’s family offers some broad brush sketches of M’s formerly independent character, and the severity of her disability. The characterisation of M by her professional carers contrasts with this evidence. In a quantity (fifty paragraphs versus sixteen) and richness that overshadows the evidence of the family, the carers paint a contrasting portrait. While disabled, M is able to communicate with noises and facial expressions (and, according to two witnesses, by speaking). She ‘makes sounds which I think is her way of telling us she wants us to do something, whether she is content or upset’,^65^ and responds to complex questions. She is childlike^66^ and innocent. Much is read into what M looks at; several staff suggest her looks express affection for particular individuals.^67^ These reports are given more colour by suggesting they indicate M’s interest in men, about which M is gently teased;

Care Worker W gave evidence of how M behaved in what she described as a “flirtatious” manner when Mr. Badwan visited. When he asked her, taking his cue from a song that was being played: “Are you a New York lady?” she pulled her arms up, pulled up her shoulders, closed her eyes, smiled, and made a two-tone noise. She has seen her behave in a similar way towards Physio L. According to Care Worker W, M seems to turn her head more and listen if a man is speaking.^68^

M engages in complex activities like watching television and responding appropriately to instructions and questions. When asked ‘what she thought of so-and-so's hair … M has responded by opening her eyes and looking at the person's hair’.^69^ M’s interest in hair underlines her connection with her past life as a hairdresser, and more, her actions are positive, contrasting to the discourse of the family that emphasises what M cannot do, and denies the meaningfulness of what she can.^70^ Her carers’ evidence stresses M’s response to music. She smiles, hums, and mouths the words to songs, becoming animated in response to some, while being reduced to tears by others.^71^ The mouthing of words, together with her contrasting emotional response to different melodies hints a rich, internalised emotional life. Her active inner existence is not confined to response to music:

M was present there while [a wedding DVD] was being played and got quite emotional, making a crying sound, although she wasn't shedding any tears.^72^

Compelling as such a portrait is, there are elements of these reports which raise some disquiet. The carers’ evidence lacks consistency—for example, only two carers report hearing speech (and neither account is corroborated). Even M’s most predominant behaviour, smiling, is not observed by her most frequent carers, C and L.^73^

Irregular reports might be explained; it is a feature of character that shows a different face to different people.^74^ Baker J acknowledges that some witnesses may have over-interpreted their evidence—indeed he also notes that a physiotherapist misrepresents hearsay as his own observations.^75^ M’s alleged interest in, and flirtation with, men lends itself most to accusations of overwrought interpretation. That M regularly avoids the gaze of others seems evidentially relatively secure. Yet this observation is often coupled with a construal of flirting^76^ that makes highly suppositional inferences about character and attitudes. Nevertheless, Baker J (with few caveats) accepts the evidence as given. Even with warnings of over-interpretation, the evidence thus retains its impact on the way the person of M is perceived. In the face of this narrative, the counter-narrative of the family recedes, while the person who cries, laughs, flirts, and plays with others is written large. The question that remains is whether such a narrative should have been introduced at all, given the power it holds and the tenuous evidence on which it is based.

### B. James: Happy and Determined

The narrative of the family in *W v M*^77^ turns on M’s autonomy, but M’s sanctity of life determines the case. This is the only case examined in this article where sanctity of life predominates. The three rulings on the case of David James^78^ again contain dichotomous claims about the identity of the patient, but this time autonomy, eventually, prevails. On their way to this conclusion the judgments indicate the dramatically different conclusions that can follow from an emphasis on either a happy and determined character, or on the burdens of treatment. The Court of Protection judgment prefers the former, simultaneously deprecating the medical narrative, while the Court of Appeal judgment favours the latter. The Supreme Court judgment modulates these narratives. While it is a hugely significant ruling, it is only of peripheral interest to my theme, and will not be discussed at length.

The case concerned withholding prospective treatments, including resuscitation and renal therapy, from critically ill David James. Mr James, a former professional musician, was in MCS, but retained the ability to interact with his surroundings, albeit at a basic level. At first instance, the hospital’s plan to limit treatment was rejected. Jackson J found that the unanimous medical evidence took insufficient account of the non-medical aspects of Mr James’ circumstances. The NHS Trust appealed and, Mr James having further deteriorated, the appeal was granted and the treatments withheld. Mr James died soon after, but an appeal to the Supreme Court was allowed. In her judgment Lady Hale P was critical of the method by which the Court of Appeal had determined the case. In particular she took issue with the Court of Appeal’s interpretation of the concept of futility, and commented favourably upon the more person-centred approach of the Court of Protection. Nevertheless, she agreed with the conclusion of the Court of Appeal, given the deterioration of Mr James by that point.

The Supreme Court judgment^79^ thus simultaneously endorsed the *verdict* of the Court of Appeal and the *approach* of the Court of Protection. In this sense it takes an emollient approach. It contains some analysis of Mr James’ character but this essentially repeats the analysis in the earlier judgments. The Supreme Court’s use of character can be best understood by analysing these earlier cases. I shall thus consider these, rather than the Supreme Court judgment.

The first decision^80^ concentrates on the interactive, family-orientated aspects of Mr James’ character. This includes a lengthy extract from the expert witness called by the Official Solicitor, describing a visit to Mr James’ bedside:

DJ showed clear signs of recognition, smiled at [his family’s] approach and mouthed what appeared to be words. He seemed to know appropriately when asked if he was feeling alright by his wife. She combed DJ's hair, during which DJ smiled. DJ was given a paper to read by his son. DJ turned the pages with his left arm.^81^

The report, while clearly indicating that Mr James suffers cognitive impairment, emphasises his interest in his family, his surroundings, and his contented nature. It is noted on three occasions in the statement that Mr James smiles, and the judgment notes this again in evidence drawn from the Official Solicitor's case manager.^82^ Five of six observations included from the medical staff^83^ relate to smiling or laughing. Evidence from Mr James’ family, as well as echoing these reports, suggests the richness of Mr James’ experience. Mr James ‘worries’ about his family,^84^ and joins in with jokes.^85^

By contrast, the medical evidence in this instance presents a detailed, but coolly technical, assessment of the past treatment failures and future treatment risks. The ability of medical evidence to provide an objective view is repeatedly challenged: evidence from the ‘impressive’^86^ Dr G dwells at length on Mr James’ frailty and poor chances of recovery. Jackson J emphasises Dr G’s admission that treatments so far had ‘worked’, including antibiotics that were given under pressure from the family.^87^ Jackson J deflects negative connotations of the diagnosis of MCS. He says Mr James’ level of consciousness ‘might more accurately be described [as] very limited rather than minimal’.^88^ Meanwhile, Dr G observes that Mr James’ awareness is ‘better with his family than with members of staff, even those with whom he is familiar’.^89^

The objectivity of medical evidence is further brought into question by the conduct of the expert witness instructed by the Official Solicitor, Dr Danbury. He states in his first report that he considers medical treatment inappropriate because, *inter alia* ‘I have collected significant evidence that leaves me with the view that DJ would prefer to be dead rather than be unable to make music’.^90^ This statement is singled out for special condemnation by Jackson J, who observes

The only basis for this last observation was a conversation with a nursing sister who says that DJ had apparently told another member of staff early in his admission to intensive care that he would prefer to die than not be able to play the guitar. Not surprisingly, DJ's family has been distressed at the use to which Dr Danbury put this snippet of information^91^

Despite the redaction of the offending comment, Jackson J indicates Dr Danbury’s credibility as a witness is fatally undermined. His amended report is not admitted as evidence of Mr James’ best interests. Yet arguably it is not clear from the judgment why such a statement should be of no interest to the court, or even subordinate to other suppositions about Mr James’ character. We might infer that second-hand reports of ‘apparent’ conversations are of low evidential weight, but this inference is not made explicit. There is no suggestion that the source lacks credibility. Rather the problem seems to be that the statement was a ‘snippet of information’ and isolated from the prevailing narrative. Great store is also set on the fact that the statement caused the family distress.

Sparing the family distress is a laudable concern, yet if the focus is on the best interests of the patient, it may not withstand the need to obtain full and frank evidence. It is also reasonable to think that an isolated remark was not indicative of Mr James’ antecedent wishes. However, it is not unreasonable to imagine that there are some things a patient may prefer to share with a health professional, but not a family member, as their distress evidences. Admitting this narrative would undermine the sense that Mr James’ close family life gives his family special insight into his likely wishes. Ultimately the court’s position seems to be that only certain indications of patient character are admissible to the assessment of best interests. Such a stance seems liable to result in quite partial assessments of the patient.

In the Court of Appeal the way the evidence is considered is ostensibly similar to the first decision. The judgment reiterates the evidence about Mr James’ character (although this time in summary), concluding ‘DJ showed his resilience and great determination to recover. … He was a remarkable man.’^92^ Despite this similarity, the Court of Appeal judgment adds extremely lengthy (they run for seven pages) extracts from the medical testimony. These emphasise the distressing side effects of Mr James’ treatment or proposed treatment. Because these detail exchanges between counsel for the NHS Trust and the medical witnesses, they are highly emotive in effect. Thus they contrast strongly with the bland list of harms and risks that characterise the earlier *NHS v DJ*^93^ and give the medical evidence dramatic prominence. For example, under questioning, Dr G explains that prolonged vasoconstrictive effects of heart stimulating medicines like adrenaline result in mummified digits:

[Mr James] has necrotic toes, he has a number of black toes which are as a result arguably or actually probably very consistently of the treatment that we've administered to him.^94^

Similarly Dr G explains that the renal therapy, which he believes is against Mr James’ best interests, causes an extreme, protracted drop in body temperature:

What we see very commonly is a shivering or cold response, that is one we try and mitigate against but we see very frequently so we induce a very unpleasant experience. … if you were to see a member of the public who is out and who is exhibiting you know clear distress from cold, shaking and so on. [This lasts] about sort of 24 hours course.^95^

Sir Alan Ward’s intention in such lengthy recounting of the medical evidence is ostensibly to inform the family,^96^ yet the effect is also to graphically emphasise the burden of treatment. In this instance then, character is relegated to a secondary role next to the side effects of intensive care treatment. Mr James, as much as he can be seen, may be resilient, but we might ask if it is fair to call on such resilience in the face of a series of grim side effects and ghastly sequelae. Thus, in these two decisions, changing emphasis from Mr James as smiling family man to critically ill patient is used to frame opposing conclusions.

### *C. Lincolnshire v N*: Independent and Resistant

Like the decisions about David James, *Lincolnshire v N*^97^ focuses predominantly on the patient’s current behaviours, drawing on character to drive a narrative about best interests. There is agreement about N’s vulnerability, but disagreement about what action is incumbent on her carers as a result.

N was a woman in her early fifties who had been in a MCS for 13 months following a brain haemorrhage. Attempts at rehabilitation had been fruitless. N had been moved to a care home, where, after 4 months, her feeding tube was found to be dislodged. The court heard that movements of N’s left arm had interfered with clinical interventions since admission. A review by a consultant neurologist concluded these movements were likely to be a response to sensory stimulus rather than volitional in nature. This notwithstanding, the movements frustrated attempts to resume ANH, and N dislodged replacement feeding tubes in a variety of ways. Replacing the feeding tube in an alternative site on N’s abdomen (that might be at less risk of dislodgement) was discounted by N’s surgeon. His opinion was that this risked the potentially fatal consequence of food accidentally entering N’s abdominal cavity if the tube, again, was dislodged. Lacking a satisfactory medical solution, the Hospital Trust applied for a declaration of the lawfulness of non-feeding. The case reached the court as an emergency, N having been without nutrition for more than a month by this time. The court heard evidence from N’s family that N would not have wished for feeding to continue, and the family strongly believed that N’s arm movements were an indication of this wish. Pauffley J held that medical treatment was no longer in N’s best interests. Nutrition was therefore been withheld and intravenous fluids withdrawn. This was due to the lack of prospects of recovery and the risks involved in resiting the feeding tube, but N’s views and her families wishes were taken into account.

N’s character is primarily mooted in evidence from N’s daughters and estranged husband. They argued that N had been a private person who would wish for her privacy to be respected. A cousin said of N’s character that ‘even when young, N did not enjoy being touched; and so the necessity of having everything done for her, as now, must be intolerable for N’.^98^ N’s daughter reported that N had said to a friend that she would ‘not like to continue life in a reduced capacity’^99^ should she be hurt in a car accident.

The family, then, suggested that N’s rejection of ANH was intentional, and consistent with both her (lifelong) private character and antecedent statements. Crucially, despite the contrary opinion expressed in the consultant neurologist’s report,^100^ the court treats the volitionality of N’s obstructive movements as an open question. Pauffley J gives significant weight to the opinion of the jointly instructed expert, Dr Jones, a consultant gastroenterologist:

[Dr Jones] formed the opinion that N's awareness of his presence was recognisable and that she did not want him to examine her. … He states that “it is possible that despite her severe cognitive impairment as part of her [MCS], she is able to express her refusal of these treatments”^101^

The diagnostic criteria for MCS include the localisation of noxious stimuli, automatic movements like scratching and purposeful reaching for objects. Yet, the severity of the cognitive disability that accompanies a diagnosis of MCS implies little understanding of the consequences of a behaviour. Analysis of the adequacy of any refusal that N’s behaviours express is missing from the judgment. Dr Jones’ evidence is persistently worded to paint N’s actions in a volitional light^102^ and Pauffley J summarises N’s clinical course in similar terms:

On admission, N was frequently resistant to physical interventions including routine observations and personal care - pushing staff away with her left hand … N shows no inclination or ability to eat or drink^103^

Pauffley J describes Dr Jones in exceptionally glowing terms: he is ‘not only a gastroenterologist of very great experience and expertise he is also an individual of enormous compassion and great insight into the human condition’.^104^ Nevertheless, Pauffley J’s reasons for preferring Dr Jones’ evidence are obscure, given Dr Jones’ opinion of N’s neurology is both outside his speciality and contradicts the evidence of the relevant specialist.

The *ratio* of the court is grounded, quite reasonably, in N’s poor prognosis and, significantly, the lack of available life-saving options. Yet N’s views, and her family’s wishes, are taken into account and it is the character and volitional elements that most strongly feature in the judgment, and underwrite a narrative of upholding patient autonomy. Behaviours of patients with disorders of consciousness are notoriously ambiguous, so this seems to be a precarious rationale.^105^ In this case, N’s current behaviours, which the court has heard are a reflexive response and, according to the most relevant expert opinion, undertaken without volition, are consistently portrayed as not only volitional, but amounting to N’s understanding that they will result in her death. Evidence from N’s family of N’s past character is used to further support this narrative. Yet to find that N’s current behaviour is a manifestation of her earlier character in order to offer a narrative of cogent treatment refusal is a radical claim. The underlying reasons for making such a claim will be examined shortly, after we have analysed our final case, *M v N*.^106^

### *D*. *M v N:* Selfish and Feisty

Late in 2015 Hayden J heard the case of *M v N,^107^* which is the final case I analyse here. In reaching the decision the judge draws on extensive testimony of the patient’s family about the patient’s past character. This draws out two traits: her selfishness, and her pugnacious approach to life.

N was a 68-year-old woman in the advanced stages of MS. N had lost capacity many years before the application, and was now agreed to be in MCS. Withdrawal of ANH was proposed in a court action that arose from her daughter’s concerns over N’s quality of life. The court heard detailed evidence from N’s family about her lack of acceptance of her diagnosis and her often violent resistance to others’ attempts to care for her. The application was unopposed by N’s clinicians, who agreed she had no prospect of recovery. Hayden J concluded that this was a matter where personal autonomy outweighed concern for the sanctity of life and granted the application to withdraw treatment.

The evidence from her family was that N had been a difficult person who coped badly with her chronic illness. The court heard that N’s difficult character had been apparent long before her diagnosis.^108^ Indeed, the evidence suggests N’s difficult behaviour caused her divorce and her alienation from her teenage daughter (who chose to live with her father after the separation).^109^ N’s challenging character, then, was deep rooted, and in full health she was allegedly vain, shallow and selfish. Her family said that they ‘all knew her as a woman for whom outward or public appearance was enormously important’.^110^ She had been dissolute, ‘“lived to shop”. [and] “loved the good life”’.^111^ Hayden J felt ‘sure that there were occasions where Mrs N rather tested [her husband] with her occasional profligacy’.^112^ She had been caustic and ‘withering and coruscating in her condemnation of people’.^113^

Once diagnosed with MS, N’s already challenging character was amplified. She had become depressed and had refused to engage with her diagnosis.^114^ She had rejected equipment provided to prevent falls, endangering her safety so she consequently suffered potentially serious accidents.^115^ She had appeared unable or unwilling to take responsibility for her own health. Her recklessness with her own well-being was mirrored in the disregard she had shown for the well-being of others. She

…was frequently in low mood, screaming, crying and hitting staff. … by March 2006 it seems Mrs. N's behaviour had escalated such that she was considered to be violent to her carers.^116^

N’s uncooperativeness, violence, and aggression alienated others. The court heard she had sustained very few close relationships.^117^

While this assessment is bleak, Hayden J also emphasises what he claims are more positive traits. He recounts N’s courageous fight for legal recognition and support for her illegitimate son, defying the moralising of her community.^118^ Further, she had adored her own parents, and was ‘heartbroken’^119^ when they were diagnosed with dementia. Yet the details of this story add to an unsympathetic portrait of N:

Mrs. N's reaction to this shocked her husband and both her children. She hated seeing [her parents] in such a diminished state … L and the children visited regularly but even under pressure Mrs. N would only go rarely. [N’s daughter] recalls her mother saying, at the time, ‘if I ever get like that shoot me!’^120^

This evidence is used by Hayden J to make a moral determination of N’s character. She was ‘capricious, selfish and seemingly shallow … immensely “proud”, jealous of her privacy, extraordinarily “feisty” … profoundly loyal to her children’^121^ Determining that such character traits give rise to a strong indication of her wishes Hayden J concludes:

[her family infer] she is a strong enough personality to take control of how she lives out the remaining period of her life. … I have no difficulty in accepting the family's view that she would not wish to continue as she is.^122^

Hayden J moulds N’s story into a plaintive cry for autonomy.^123^ Yet, perhaps more than any of the cases considered here, the way character is used inspires disquiet. N’s behaviour and diagnosis invites an analysis that puts autonomy onto a more questionable footing. There is a known association between MS and a range of psychiatric conditions,^124^ and neurological changes associated with MS may result in pathological personality changes.^125^ That MS may have manifested in disturbances of N’s mind seems to have been considered, at least in passing, by Hayden J who observes:

It may be that for sometime prior to her diagnosis the disorder had begun to have its effect on her general functioning. … Within six years of diagnosis she became wheel chair dependent, struggled with concentration, experienced rapid mood changes and difficulties with her memory.^126^

N’s purportedly disagreeable character is well documented and precedes her diagnosis of MS. It is impossible to say with any certainty whether these character traits themselves were pathological. Nevertheless, if N’s behaviours were symptoms of an underlying disease process, it shows a remarkably facile approach to mental illness to link these behaviours to autonomy, since the presence of a mental illness raises fundamental questions about N’s agency. But perhaps this goes too far. The presence of MS suggests that there was no treatment available that would have arrested N’s antisocial behaviours in the long term. Finding that the effects of MS may have altered N’s reasoning is therefore unlikely to have altered the final determination of best interests in this case. Nevertheless, we should be suspicious of a narrative designed to champion autonomy.

Even without such supposition there is a greater question lurking here. All of these cases could be solved less problematically by looking to the wishes of the family or the futility of treatment— indeed, the latter frequently features in the *ratio*. We might explain the discussion of character in these judgments as a simple reassurance to families. By suggesting that the outcome is ‘what the patient would have wanted’, we address the burden of responsibility or distress that families may otherwise carry. Yet the discussion of character is too frequent and extensive for this to be a wholly satisfactory explanation. The way it is used in these judgments maps onto particular philosophical perspectives of well-being. These may resemble accounts that say what we desire is what is best for us, although this account is particularly problematic when employed in instances of antecedent decision-making.^127^ This notwithstanding, the courts arguably appear to be doing something else. Instead of simply giving expression to autonomy and choice, the courts appear to be uniquely concerned with using narratives of character to facilitate autonomy, regardless of whether autonomy is desired by the patient. This suggests that the law, rather than allowing self-expression, says that self-expression is a, or perhaps *the*, primary property of being human. I will explore these philosophical perspectives now.

## IV. THEORETICAL BASES OF CHARACTER EVALUATION AND WELL-BEING

In all of these cases character plays a prominent role in the determination of well-being. While best interests are held in common law to encompass a plethora of medical, emotional, and other interests, an increasingly narrow focus on antecedent desire suggests inconsistencies between this approach and the autonomy focus of best interests in the MCA 2005. This inconsistency may be driven by a determination to increase the role of substituted judgement in deciding best interests.^128^ By using the patient’s character to determine their best interests, there is *prima facie* a close link between this approach and desire accounts of well-being. To remind ourselves, these accounts suggest that a person’s life will go best if we satisfy their preferences. They are ‘probably the dominant view of welfare among economists, social scientists, and philosophers’.^129^ Yet closer examination of MCS cases suggests a more sophisticated understanding may be necessary, for the facts of the cases present multiple problems for a desire account.

An unembellished desire account might suggest that satisfaction of any desire at any time enhances welfare. To understand the problems of applying desire accounts to MCS, we need to understand that few (if any) theorists defend such an account. Most desire-theorists specify significant caveats. These include that welfare-enhancing desires should: be fully informed (either using ideal outcomes or by considering all possible personal effects of a desire); be correctly motivated (not sordid, or mischievous, or inane); come into effect at a time when I exist (not after I die); and enter my awareness.^130^ Given the scale of the incapacity of the patients in the cases I have considered, there seems little chance that their desires can match these criteria.

Antecedent desires concerning treatment refusal (in the form of an ADRT or an LPA) may represent a realistic compromise,^131^ especially if they satisfy at least some of these criteria. Yet none of the descriptions of antecedent wishes in these cases come anywhere near to doing so, nor are they engaged under the current legal framework. Indeed, at most the cases contain impressionistic sketches of the patient’s values. By directing these sketches towards actual decisions, the law seems to be doing something additional to, or even different from, establishing antecedent desire. Rather it seems to be saying that to possess these antecedent desires is a necessary precondition to our well-being.

The fervent commitment of the law to facilitating the autonomy of the patient despite any counterfactuals suggests a fetishisation of autonomy that goes beyond a desire account of well-being. It seems much closer to an ‘objective list’ account of well-being,^132^ which offers a simple list of the states of affairs that are necessary for well-being that is separate from a person’s desires or pleasures. Specifically, the approach in these cases resembles an objective list account of well-being where autonomy is a primary (and in some cases *the* primary) item. As I will explain below, this approach resembles a specific type of objective list account named the ‘perfectionist’ account of well-being.

### A. Perfectionist Accounts of Well-Being

To recap, objective list accounts of well-being contain lists of states of affairs that are good for people, whether or not they desire them or derive pleasure from them.^133^ Objective lists commonly contain items relating to moral goodness,^134^ rationality,^135^ aesthetic awareness,^136^ and friendship,^137^ yet usually lack coherent explanation of *why* these items should be included. They suffer the criticism that they are arbitrary and lack the theoretical depth to explain why the items they list are important or how they should be weighed.^138^ In order to provide this theoretical depth, perfectionist accounts look to theories of what constitutes a virtuous life. Theories of the virtuous life are drawn from *virtue ethics*.^139^ Put simply, this is based on the Aristotelian view that things should have ‘virtues’ (αἱ ἀρεταί) to be good examples of what they are. Certain human virtues are related to moral character. These moral virtues are part of the fabric of the individual: who we are, not what we do, marks moral excellence. Scholars in the virtue ethics tradition argue that human virtues can be identified by observing human conduct, language, and psychology.^140^ Accounts of virtue are diverse but common accounts include virtues such as truthfulness, courage, and non-malevolence.^141^

According to perfectionist accounts, these virtues can be used to identify the natural human capacities (such as rationality and autonomy) that are necessary to live a virtuous life.^142^ As one account has it, virtues arise from human nature; we can identify certain capacities that all humans need in order to have a virtuous existence, and populate an objective list with these capacities.^143^ Exercising these capacities in a virtuous way will then allow a person to live a virtuous existence. Arguably this describes an objective list account of well-being with a firm theoretical grounding.^144^ Perfectionist accounts generally suggest the capacities necessary for human well-being centre upon basic levels of health, some form of rationality, and moral autonomy.^145^ The prospects for the well-being of a patient in MCS might appear slender, since, however their health is perceived, they are capable of exercising neither rational nor moral autonomy. On one hand then, a perfectionist account may suggest that even a putatively content patient like David James^146^ lacks the fundamental capacities to achieve well-being. However, when faced with a person whose capacities are obstructed, we might instead attempt to promote their well-being by fostering these capacities. If a patient were rational and autonomous, but unwell, it would be uncontroversial to try to heal them. Therefore, to return to the example of David James, we might promote his rationality and his moral autonomy to allow foster his well-being. I suggest we can shelve any appraisal of the former (here), given the requisite levels of rationality are arguably implicit in judicial decisions. Instead, let us focus on autonomy.

Promoting moral autonomy, despite the roots of this account in virtue ethics, does not require a judge to make a final judgment of the patient’s virtue (although, as we see in *M v N*,^147^ such sentiments may follow). Instead they need only appeal to a core account of human capabilities that are necessary to achieve well-being. Such a claim clearly coincides with the emphasis on autonomy within the MCA 2005. A perfectionist account of well-being manifests in the following way: where the wishes of the incapacitated patient are not clear, best interests are engaged in line with section 4.6(b). A determination of the beliefs and values of the patient is made according to the patient’s character. The patient in a MCS is assumed to have cogent wishes, but to lack the ability to act upon them. As the cases in our series demonstrate, a judge needs only search for these wishes in the patient’s character and supply the means by giving effect to these wishes. This allows the patient in MCS to exercise this autonomous capability according to their own ends. If they wish, the judges (and thus the liberal state) may remain scrupulously neutral about whether such an end is virtuous.

## V. CRITICISMS OF PERFECTIONIST THEORIES OF WELL-BEING IN THE LAW

Identification of this theoretical underpinning provides several grounds for criticism of the law. For example, critics of perfectionist approaches suggest the claim that an objective list can be created from an account of human nature is fallacious, because the concept of human nature is innately problematic.^148^ In terms of the MCA 2005 this raises questions about the value given to autonomy in its application: even if autonomy is a generally valid basis on which to make decisions, there may be exceptions.^149^ In the context of MCS we could suggest autonomy occupies too dominant a position, risking drowning out other important factors, such as social relationships. Further questions arise if we critically examine the theoretical bases of perfectionist approaches, particularly their connection to the ‘common-sense’ approach to character found in virtue ethics. More enlightened approaches to character raises questions about the degree to which character can be understood within a narrative structure, especially in an adversarial system of law. Evidence from the psychological literature further supports these concerns, especially the validity of understanding character as composed of enduring traits. The final part of this article considers these criticisms in turn.

### A. Autonomy

The importance of autonomy in medical jurisprudence is well established, and apparently increasing.^150^ There is very widespread agreement that respect for autonomy is ethically important:^151^ its diffusion into medical practice has been argued to be the major achievement of bioethics.^152^ Where the patient lacks capacity, an advance decision or an LPA, our cases suggest the Court of Protection may give expression to a patient’s autonomy using evidence from the patient’s character. While this article lacks the scope to ground a general criticism of autonomy, giving such weight to autonomy in cases involving MCS raises questions about the consistency of autonomy as a concept in the law. As autonomy is far from being a single, unified, concept much depends on the conception of autonomy that is being deployed and this is often opaque. The Kantian conception of autonomy suggests an individual requirement to exercise rational agency, and emphasis on capacity to make decisions in the MCA 2005 speaks most readily to this approach.^153^ The conception of autonomy attributed to J.S. Mill is more suggestive of a freedom to live as one wishes that relates to the protection of the liberty of minorities.^154^ An increasing emphasis on Millian liberty is detectable in the desire to extend equality to the vulnerable and voiceless in recent Court of Protection cases, including, significantly, *M v N.*^155^ Thus, these cases suggest conceptual inconsistencies between statutory and common law interpretations of autonomy. At a minimum, this may cause judgments to be unpredictable, with all the problems this implies.^156^

Even ignoring these inconsistencies, the appropriateness of autonomy to the circumstances of MCS cases seems questionable. Critique of the ethic of autonomy highlights its overshadowing of the value that people place on interpersonal relationships.^157^ Others contend an emphasis on autonomy denigrates the essentially caring nature of patient–professional relationships in healthcare.^158^ Such criticisms seem especially cogent here, despite the sensitivity Judges in our case series undoubtedly showed to relational issues. Such sensitivity could conceivably give voice to relationality and care while emphasising autonomy by expressing, for example, autonomous wishes that others decide on one’s behalf. Yet the predominant approach in the case series was to craft family or professional narratives into expressions of autonomy. Since many patients will be in MCS following sudden, acute events, planning for this event is likely to be rare.^159^ In the absence of an ADRT or LPA, by seeking an autonomy-based narrative based on character judges risk overlooking the great variety of human impulses regarding impending death, including patients having no plans whatsoever. One typology suggested the forty-two residents of four Hong-Kong care homes demonstrated at least five different approaches to end-of-life decision-making.^160^ Some residents wanted treatment at any cost. Others variously favoured independence, making a judgment based on the burdens of treatment, trusting in fate and trusting their doctors. This hints at a multiplicity of approaches to death that we may overlook by emphasising autonomy-based narratives of a good death in every circumstance.

### B. The Weakness of Narrative

If a fixation on using the lens of autonomy to view human nature is concerning, the suitability of the courts as a venue for such work also raises questions that are quite separate from those raised by autonomy. Adversarial law tends to frame facts in partial ways, seeking a relatively solid and homogenous view of the patient’s wishes, feelings, beliefs and values. It is appealing to argue, as judges frequently do, that those closest to us know us best and can offer important reflections on our interests. This understanding of the self is vulnerable to the established view that the self is essentially socially constructed. As Mead observes:

We carry on a whole series of different relationships to different people. We are one thing to one man and another thing to another. There are parts of the self that exist only for the self in relationship to itself. We divide ourselves up in all sorts of different social interactions. It is the social process itself that is responsible for the appearance of the self^161^

Such a view is, broadly speaking, accepted in contemporary understandings of the self.^162^ The implication is not only that we must canvass widely to understand the self, but also that the self is not itself truly apprehensible to anyone other than the subject. Such a position, while upholding the preference for an advance decision in MCA 2005 at section 4(6)(a), raises questions about how we might gain the knowledge sought by section 4(6)(b) in the absence of such a decision with any degree of certainty. Certainly, the adversarial nature of common law encourages particular views of persons and events, suggesting it is an unsuitable arena to collect the multitude of narratives that might offer any glimpse of the person ‘in the round’. Adversarial law, in its search for narratives, will make choices about the way evidence is presented. The partiality of judicial narratives in common law has long been understood, especially, although not exclusively,^163^ in the criminal law.^164^ There, scholars have noted the way that narratives are shaped and moulded according to the needs of protagonists:

form of argument, selection of witnesses and evidence is governed by counsel’s need to present a plausible account … the judge adopts [similar] methods of argument^165^

Adversarial law implies a need to select and favour evidence in order to devise a compelling, unfolding narrative, while discrediting elements contrary to that narrative. It may be protested that differences between criminal and civil law imply less adversarial approaches in MCS cases. Nevertheless, the ability of the civil courts to construct partial narratives has been noted elsewhere,^166^ and seems amply made out by the differences between the focus of the Court of Protection and Court of Appeal in *James*. Even in a potentially diminished form, adversariality is clearly an impediment to using narrative to furnish a robust description of character. Instead, such a description must seek as many narrative threads as possible, canvassing widely among those who knew the person if it is to do justice to the dynamic nature of the self.^167^

### C. Character in Psychology

Judges must determine best interests, and to do so means determining—or perhaps constructing—the wishes and beliefs of the patient. In the cases we have examined we have seen these wishes and beliefs are based on a narrative that draws heavily on character. I have questioned the emphasis on autonomy in the circumstances of MCS, and suggested that the adversarial nature of the law may lead us to overly partial narratives. As I shall discuss now, these problems are multiplied by the weakness of character as a predictor of decisions.

The majority of contemporary personality psychology follows the ‘five-factor’ model. This proposes that personality traits can be measured along five continua.^168^ One of the major criticisms of this approach is that individuals do not consistently reproduce personality traits when they are measured repeatedly across different situations.^169^ The seminal study of this issue^170^ measured honest and dishonest behaviour in 8,000 schoolchildren. It found that the level of correlation between a child’s (dis)honest behaviours and the situations in which their honesty was tested was extremely low. For example, children who readily cheated when given the opportunity to inflate written test scores did not reliably do the same when given opportunities to cheat when completing a puzzle. The problem has been overcome by aggregating behaviours over sustained periods^171^ in order to produce a ‘dominant’ tendency. Yet this method is both controversial^172^ (since contrary traits that are in the minority are ignored) and at best concedes that character can be measured only in general terms.

These seminal experiments looked at the behaviours of children, who might be expected to display more erratic behaviours than adults. Moreover, the MCA 2005 seeks to guide decision-making in extremely difficult circumstances. Even accepting that deviation from normal behaviours occurs, dominant characteristics may be argued to be accurate enough for the situations in which best interests must be determined. However, it is these very situational factors which give rise to a second indication that character may be of poor predictive value, and this can be observed whatever the age of the person in question.

Psychologists of the ‘situationist’ school argue that character is a far weaker driver of behaviour than circumstantial factors. They point to a large number of behavioural experiments where people act in ways that seem directly influenced by situational cues. In one experiment, users of a public telephone were given the opportunity to help an experimental confederate who dropped a sheaf of papers. Helping behaviour strongly correlated with whether the test subject had unexpectedly found a dime in the telephone box directly before the encounter.^173^ Another experiment measured the helping behaviour of seminary students. On their way to deliver a sermon, the students had a confected encounter with an experimental confederate who was slumped in a doorway in a feigned state of distress. Helping behaviours correlated with whether the students had been told prior to setting out that they were running late or early.^174^ Internationally, a huge number of studies have reproduced Milgram’s ethically controversial experiments on obedience.^175^ These measure the compliance of test subjects in delivering (faked) electric shocks to an experimental confederate on the orders of a white coated ‘scientist.’ The results of these experiments were remarkably uniform across cultures, ages and genders. They indicated in almost every instance that two-thirds of test subjects, despite marked personal duress, were fully obedient to the scientist’s polite but firm instructions, even when they received cues that the confederate had been (perhaps fatally) injured by the voltage.^176^ Situationists have argued that, even if enduring character traits exist, these experiments indicate that even small changes in situational factors can radically influence behaviour.^177^ It may be objected that many of these experiments took place many decades ago, before the negative impact of research involving deception and duress upon the participants was widely acknowledged. Nevertheless the abundance of such experiments and the consistency of the results they produced suggests we should give these findings credence, despite their age and ethical deficiencies.

Evidence from experimental psychology raises questions about the consistency of dispositions and suggests that consistent dispositions do not result in consistent behaviour. It also indicates the important role of situations in determining behaviour. By doing so, it damages the ‘common-sense’ approach to character as an enduring, predictable, and dominant feature in decision-making, on which the law apparently relies. In particular, character and disposition seem unreliable if we try to predict specific behaviours in specific situations. Yet this is exactly what the courts are wont to do in the cases described.

## VI. CONCLUSION

Our preliminary investigation into the interaction between bioethics and medical law used a thematic approach to analysing judgments of the best interests of patients in MCS. A number of themes arose from the analysis, including (in many cases) the frequent use of narratives about the character of patients.

I have analysed some of these cases above. In *W v M*,^178^ the present and past character of the patient was contended. In successive instances of *James*,^179^ differing emphasis was placed on the determined and family-orientated character of the patient or on their extreme fragility. In *Lincolnshire v N*,^180^ character was used to bulwark an interpretation of a patient’s actions. In *M v N,^181^ a* sustained discussion of character was undertaken. In all but one of these cases (*W v M*), character was used to furnish an autonomy-facing narrative. Also noted, but not explored, were the frequent characterisations of the family and other witnesses in these judgments. At a basic level, the use of character might be explained in the latter by the need to determine a witness’ credibility. In the case of patients themselves, it speaks of the requirements of the MCA 2005, section 4(6) to determine the values, beliefs, wishes, and feelings of the patient. How better to do this than to investigate character? Indeed, anecdotally, clinicians aver this is as a common approach in best interests meetings in the clinic. At a deeper level this may indicate the direction of travel of wider health law. The recent advent of the ‘particular patient standard’ in the law of consent^182^ could conceivably mean that doctors must engage with their patients’ fundamental personal motivations. Such an approach may involve an exploration of patient character and narrative.

The use of character to focus exclusively on expressions of autonomy accords with a perfectionist theory of well-being. Perfectionist approaches populate an ‘objective list’ of goods according to the human capabilities that are needed to live (and die) virtuously. In this reading of the law, the MCA 2005 at section 4(6) declares the capability of autonomy to be essential to having a good life and death. The character of patients is used to furnish decisions within this category of autonomy, with the judge supplying the agential means for the patient to express their character according to their own ends. While this approach appears sympathetic to the pro-autonomy inclination of the MCA, it raises significant concerns about the consistency and comprehensiveness of an understanding of human nature that accords autonomy such a dominant role. Given the potential for autonomy to be used to express both Kantian rationality and Millian liberty, it may lead to inconsistent judgments. Moreover, while autonomy may be used to express a patient’s wishes about the weight they want the opinions of their loved ones or doctors to have in deciding their treatment, it seems a poorly adapted vehicle for this task, and suggests too little attention is paid to relational or care-based accounts of healthcare decision-making. The identification of a perfectionist approach also indicates that questions need to be asked about the understanding of character on which these judgments are based. Adversarial law requires clear narratives that discredit contradictory reports. This makes the court a poor arena for a full and expansive exploration of the patient’s character. It has long been recognised that a person can have many, sometimes contradictory, facets—indeed some of these may never be shared with others. Insights from experimental psychology further undermine the worth of evaluations of character, for moral (and other) traits are inconsistently exhibited and easily influenced by situational factors. These insights challenge the folk-wisdom account of character upon which the law apparently rests.

What, then are we to do when faced with patients whose severity of illness is so great that we doubt it should be borne? Ultimately, the range of concerns I have raised suggests the patients in these cases are simply beyond the limits of autonomy. Indeed, even when aware of approaching death, I have suggested that some people may favour other approaches, such as putting their trust in families or clinicians to do the right thing.

Despite these reservations, we may nevertheless still wish to make some determination of what a person may have wanted. Making decisions on behalf of others is morally burdensome. It may be more than a family, clinician, or judge can bear to make an extremely weighty decision without some sense it would be agreed to by the patient. In these cases we must recognise both the limitations of an adversarial approach and the inconsistency of character. Better techniques may exist, and an engagement with, and research into, narratological approaches to ethics may be needed to raise the calibre of current approaches to best interests decision-making.^183^ Above all, we must recognise that, in part, we make these inquires for our own benefit. This itself speaks most loudly to approaches that favour relational and care ethics, and suggests we must be wary of using justifications based on autonomy that have little grounding in psychological understandings.

I recognise that a critique of character implicitly challenges many of the assumptions that underlie, not just the workings of law, but also society itself. Character evaluation is basic human currency. We marry, make friendships and workplace alliances, feel socially supported or undermined, all on the basis of the predictable and enduring nature of character. Yet in all of these instances, our behaviours result from a social bargain in which we have some control and agency. With significant reservations, we can conclude that autonomy (sort of) works. Yet there are limits to autonomy. We deceive ourselves if we do not admit it in these cases.

## Footnotes

1 P Collier, *Sermons* (EP Dutton 1892) 216.

2 JA Holstein and JF Gubrium, *The Self We Live By: Narrative Identity in a Postmodern World* (OUP 2000).

3 J Doris, *Lack of Character: Personality and Moral Behaviour* (CUP 2002).

4 Eg J Winter, ‘The Truth Will Out? The Role of Judicial Advocacy and Gender in Verdict Construction’ (2002) 11 S & LS 343.

5 MCA 2005, ss 24–26 and 9–14, respectively.

6 D DeGrazia, ‘Value Theory and the Best Interests Standard’ (1995) 9 Bioethics 50.

7 Perfectionist theories of well-being should not be confused with other theoretical guises of perfectionism, eg political perfectionism.

8 G Bradford, ‘Perfectionism’ in G Fletcher (ed), *The Routledge Handbook of Philosophy of Wellbeing* (Routledge 2016) 124.

9 JT Giacino and others, ‘The Minimally Conscious State: Definition and Diagnostic Criteria’ (2002) 58 Neurology 349.

10 Court of Protection Rules, Practice Direction 9E, para 5. At the time of publication this approach may be evolving*.* See*: M v A Hospital* [2017] EWCOP 19.

11
*Airedale v Bland* [1993] AC 789.

12
*Frenchay v S* [1994] 2 All ER 403; *Re D* [1997] 38 BMLR 1; *Re H* [1998] 3 FCR 174; *A v H* [2002] 1 FCR 713.

13
*Bland* (n 11).

14 [2011] EWHC 2443.

15
*Aintree v James* [2013] UKSC 67.

16 [2016] EWCOP 53. The *Briggs* decision was delivered during the writing of this article and does not form part of this analysis, although it appears consistent with it.

17
*Bland* (n 11).

18
*W v KH* [2004] EWCA 1324.

19 MCA 2005, s 25(5–6).

20
*Re A (Male Sterilisation)* [2000] 1 FCR 193, 200 (Butler-Sloss P).

21
*Bland* (n 11); *W v M* (n 14); *James* (n 15).

22
*Bland,* ibid; *James*, ibid.

23 Eg *Briggs* (n 16).

24 J Baker, ‘A Matter of Life and Death’ (2017) 43 J Med Ethics 427.

25 Eg E Wicks, ‘When Is Life Not in Our Own Best Interests? The Best Interests Test as an Unsatisfactory Exception to the Right to Life in the Context of Permanent Vegetative State Cases’ (2013) 13 Med L Int 75.

26 Eg M Donnelly, ‘Determining Best Interests under the Mental Capacity Act 2005’ (2011) 19 Med L Rev 304.

27 Eg LM Kopelman, ‘The Best Interests Standard for Incompetent or Incapacitated Persons of All Ages’ (2007) 35 J Law Med Ethics 187.

28 D Parfit, ‘Reasons and Persons’ (OUP 1987); DeGrazia (n 6).

29 V Tiberius, ‘Prudential Value’ in I Hirose and J Olson (eds), *The Oxford Handbook of Value Theory* (OUP 2015) 158.

30 MD Adler, ‘Value and Cost-Benefit Analysis’ in Hirose and Olson, ibid 317; E Angner, ‘Well-being and Economics’ in Hirose and Olson, ibid 492.

31 Tiberius (n 29).

32 G Fletcher, ‘Objective List Theories’ in Fletcher (n 8) 148.

33 ibid*.* There is a debate as to whether objective lists can contain only one item.

34 Parfit (n 28) 499.

35 Fletcher (n 32).

36 As such, this paper forms part of a wider project (see below) which has so far published two linked articles on this theme: R Huxtable, ‘From Twilight to Breaking Dawn? Best Interests, Autonomy, and Minimally Conscious Patients’ (2016) 24 Med L Rev 622; R Huxtable and G Birchley, ‘Seeking Certainty? Judicial Approaches to the (Non-)Treatment of Minimally Conscious Patients’ (2017) 25 Med L Rev 428. A third paper looking at the ethical values within MCS judgments is planned.

37 Huxtable and Birchley, ibid.

38 Databases were searched for cases in December 2015 and July 2016. The cases were: *Frenchay* (n 12); *Re D* (n 12); *Re H* (n 12); *A v H* (n 12); *W v KH* (n 18); *W v M* (n 14); *Re JD* [2012] EWHC 4420; *NHS Trust v L* [2013] EWHC 4313; *James* (n 15); *NHS v VT* [2014] COPLR 44; *Sheffield v TH* [2014] EWCOP 4; *County Durham v PP* [2014] EWCOP 9; *Lincolnshire v N* [2014] EWCOP 16; *Gloucestershire v AB* [2014] EWCOP 49; *St George’s v P* [2015] EWCOP 42; *M v N* [2015] EWCOP 76; *Re S* [2016] EWCOP 32. Since the search further cases have been decided: *Briggs* (n 16); *Abertawe v RY* [2017] EWCOP 2; *M v A Hospital* [2017] EWCOP 19.

39 J Thomas and A Harden, ‘Methods for the Thematic Synthesis of Qualitative Research in Systematic Reviews’ (2008) 8 BMC Med Res Methodol 45.

40 While the identification of facts may be clear to a legal audience, the scrutiny of values and approaches may require more explanation. Values were identified either because they were explicit (eg naming autonomy or the sanctity of life) or implicit (ie a pro-autonomy position might be made out in discussion of dependence) in each judgment. The analytical technique allowed clusters of similar statements of value to be collected into themes that could be scrutinised for philosophical clarity and coherence. Approaches were similarly either explicit (did the judge draw up a balance sheet or not?) or could be made out implicitly, eg by the degree of influence family versus professional witnesses appeared to have on the judges reasoning. Examples of the latter can be found in Huxtable and Birchley (n 36).

41
*W v KH* (n 18).

42
*M v N* (n 38).

43 Huxtable and Birchley (n 36).

44
*M v N* (n 38).

45
*Sheffield v TH* (n 38).

46 The only case where character did not appear was *A v H* (n 12).

47 D Nicholson, ‘Telling Tales: Gender Discrimination, Gender Construction and Battered Women Who Kill’ (1995) 3 Fem LS 185; Winter (n 4); EN Yankah, ‘Good Guys and Bad Guys: Punishing Character, Equality and the Irrelevance of Moral Character to Criminal Punishment’ (2004) 25 Cardozo L Rev 1019.

48 Nicholson ibid.

49 ibid 190.

50 Doris (n 3) 128.

51
*Re H-B* [2015] EWCA Civ 389, [75–76].

52
*St George’s v P* (n 38).

53
*County Durham v PP* (n 38).

54 W McDougall, ‘Of the Words Character and Personality’ (1932) 1 J Pers 3; A nuanced account of such distinctions is found in: CB Miller, ‘Character and Moral Psychology’ (OUP 2014) 3–37.

55 PL Hill and BW Roberts, ‘Propositions for the Study of Moral Personality Development’ (2010) 19 Curr Dir Psychol Sci 380.

56 I also note that our discussion collides concepts of character with considerations of self-identity, which draws in other distinct literatures.

57 For example, patient character is absent in *Re H* (n 12) and *A v H* (n 12) and is mentioned only in passing in and *W v KH* (n 18), *Re JD* (n 38), *County Durham v PP* (n 38) and *Gloucestershire v AB* (n 38).

58
*W v M* (n 14).

59 ibid.

60 MCS was diagnosed in *A v H* (n 12), but the court did not distinguish this from PVS.

61 Detailed discussion of whether the person who has lost capacity is different from that person prior to their loss of capacity would be digressive. See: T Hayes, ‘A (social) Room with a View (to the Future): Advance Decisions and the Problem of Personhood’ in R Huxtable and R ter Meulen (eds), *The Voices and Rooms of European Bioethics* (Routledge 2015) 87–102.

62 It is germane to note that M’s prior wishes are weighed very lightly, while characters of family members are called into question (however sympathetically). Eg *W v M* (n 14) [129, 166, 167].

63 ibid [118].

64 ibid [116].

65 ibid [126].

66 ibid [172].

67 ibid [160].

68 ibid [171].

69 ibid [128].

70 ibid [108].

71 ibid [154].

72 ibid [161].

73 ibid [139, 144].

74 ibid [174].

75 ibid [154].

76 ibid [138, 143 and 171].

77 ibid.

78
*James* (n 15) and its earlier instances *NHS v DJ* [2012] EWHC 3524 and *NHS v James* [2013] EWCA Civ 65.

79
*James*, ibid.

80
*NHS v DJ* (n 78).

81 ibid [33].

82 Ibid [34].

83 Ibid [35].

84 Ibid [61].

85 ibid [66].

86 ibid [47].

87 ibid [44–45]; also consider [64, 67].

88 ibid [38].

89 ibid [42].

90 ibid [57].

91 ibid [58].

92
*NHS v James* (n 78) [13].

93
*NHS v DJ* (n 78).

94
*NHS v James* (n 78) [22].

95 ibid [23].

96 ibid [26].

97
*Lincolnshire v N* (n 38).

98 ibid [32].

99 ibid [30].

100 ibid [20].

101 ibid [37].

102 ibid [35].

103 ibid [13n14].

104 ibid [62].

105 RJ Jox and others, ‘Diagnosis and Decision Making for Patients with Disorders of Consciousness: A Survey among Family Members’ (2015) 96 Arch Phys Med Rehabil 323.

106
*M v N* (n 38); For alternative commentary originating from this project: Huxtable (n 36).

107 ibid.

108 ibid [4].

109 ibid [5],[53].

110 ibid [52].

111 ibid [52].

112 ibid [53].

113 ibid [6].

114 ibid [4],[10].

115 ibid [8–10].

116 ibid [10].

117 ibid [6].

118 ibid [51].

119 ibid [57].

120 ibid [55].

121 ibid [50].

122 ibid [59–60].

123 See also *Sheffield v TH* (n 38).

124 RJ Siegert and DA Abernethy, ‘Depression in Multiple Sclerosis: A Review’ (2005) 76 J Neurol Neurosurg Psych 469

125 RH Benedict and others, ‘Personality Disorder in Multiple Sclerosis Correlates with Cognitive Impairment’ (2001) 13 J Neuropsych Clin Neurosci 70; LB Strober and others, ‘Unemployment in Multiple Sclerosis: The Contribution of Personality and Disease’ (2012) 18 J Mult Scler 647.

126
*M v N* (n 38) [4–6].

127 Hayes (n 61).

128 I thank my anonymous peer-reviewer for this observation.

129 W Shaw, ‘Contemporary Ethics: Taking Account of Utilitarianism’ (Blackwell Publishing 1999) 53.

130 C Heathwood, ‘Desire Fulfilment Theory’ in in Fletcher (n 8) 135.

131 Whatever their philosophical validity. Justifying antecedent wishes on the basis of a desire account raises many objections eg whether changes in our bodies and/or minds result in us becoming, to all moral purposes, different people. See Parfit (n 28) 219–43; Hayes (n 61).

132 Fletcher (n 32).

133 ibid.

134 G Fletcher, ‘A Fresh Start for the Objective List Theory of Well-being’ (2013) 25 Utilitas 206; Parfit (n 28) 499.

135 Parfit, ibid.

136 ibid; J Finnis, *Natural Law and Natural Rights* (Claredon Press 1980); M Murphy, ‘Natural Law and Practical Rationality’ (CUP 2001).

137 Finnis, ibid; Fletcher (n 134); Murphy, ibid.

138 B Bradley, *Well-being and Death* (OUP 2009).

139 Virtue ethics is a contemporary reinvigoration of Aristotelian ethics. See GEM Anscombe, ‘Modern Moral Philosophy’ (1958) 33 Philosophy 1; for critique, see Doris (n 3).

140 R Hursthouse and G Pettigrove, ‘Virtue Ethics’ in EN Zalta (ed), *The Stanford Encyclopedia of Philosophy *(Winter 2016 edn) <https://plato.stanford.edu/archives/win2016/entries/ethics-virtue/> accessed 15 August 2016.

141 Miller (n 54).

142 D Dorsey, ‘Three Arguments for Perfectionism’ (2010) 44 Noûs 59; A Baril, ‘Virtue and Wellbeing’ in Fletcher (n 8) 242.

143 Bradford (n 8).

144 Eg S Darwall, ‘Welfare and Rational Care’ (Princeton University Press 2002); M Lebar, ‘Good for You’ (2004) 85 Pac Philos Q 195.

145 ibid*;* Bradford (n 8).

146
*James* (n 15).

147
*M v N* (n 38).

148 Dorsey (n 142).

149 P Bielby, ‘The Conflation of Competence and Capacity in English Medical Law: A Philosophical Critique’ (2005) 8 Med Health Care Philos 357.

150 Eg *Montgomery v Lanarkshire* [2015] UKSC 11; *Wye Valley v B* [2015] EWCOP 60; *Re C* [2015] EWCOP 80.

151 D Archard, ‘Informed Consent: Autonomy and Self-Ownership’ (2008) 25 J Appl Philos 19.

152 JF Childress, ‘The Place of Autonomy in Bioethics’ (1990) 20 Hastings Cent Rep 12; R Gillon, ‘Ethics Needs Principles - Four Can Encompass the Rest - and Respect for Autonomy Should Be "First Among Equals”’ (2003) 29 J Med Ethics 307.

153 J Coggon and J Miola, ‘Autonomy, Liberty, and Medical Decision-Making’ (2011) 70 CLJ 523.

154 Archard (n 151).

155 ‘to superimpose what the Court thinks best, may result in indirect discrimination. The central objective is to avoid a paternalistic approach and to ensure that the incapacitous achieve equality with the capacitous’. *M v N* (n 38) (Hayden J) [30]; Also *Wye Valley v B* (n 150) [11].

156 Huxtable and Birchley (n 36).

157 A MacLean, ‘Autonomy, Informed Consent and Medical Law: A Relational Challenge’ (CUP 2009); MA Verkerk and others, ‘Where Families and Healthcare Meet’ (2015) 41 J Med Ethics 183.

158 MacLean, ibid; A Mol, *The Logic of Care: Health and the Problem of Patient Choice* (Routledge 2008).

159 J Kitzinger and C Kitzinger, ‘The “Window of Opportunity” for Death after Severe Brain Injury: Family Experiences’ (2013) 35 Sociol Health Illn 1095.

160 HY Chan and SM Pang, ‘Readiness of Chinese Frail Old Age Home Residents Towards End-of-life Care Decision-making’ (2011) 20 J Clin Nurs 1454.

161 GH Mead, *Mind, Self and Society* (University of Chicago Press 1934).

162 Holstein and Gubrium (n 2).

163 Eg D Cowan and E Hitchings, ‘Pretty Boring Stuff': District Judges and Housing Possession Proceedings’ (2007) 16 Soc Leg Stud 363.

164 Winter (n 4); Nicholson (n 47).

165 Winter, ibid 344, 347.

166 Cowan and Hitchings (n 163).

167 Holstein and Gubrium (n 2).

168 JM Digman ‘Personality Structure: Emergence of the Five-Factor Model’ (1990) 41 Ann Rev Psychol 417; ER Thompson and GP Prendergast, ‘The Influence of Trait Affect and the Five-factor Personality Model on Impulse Buying’ (2015) 76 Pers Individ Dif 216.

169 H Hartshorne and M May, *Studies in the Nature of Character, Vol I : Studies in Deceit* (Macmillan 1929); The studies measured a correlation coefficient of 0.23 (0.1 is near meaningless and 0.9 is strongly correlated). Also see: W Mischel, *Personality and Assessment* (John Wiley and Sons 1968). Further similar studies by Mischel of various populations rarely produced correlation coefficients of above 0.3, suggesting accurate prediction of responses of particular people to particular situations is unlikely.

170 ibid.

171 Correlation coefficients of 0.86 have been measured this way. See: S Epstein and EJ O’Brien, ‘The Personal and Situation Debate in Historical and Current Perspective’ (1985) 98 Psychol Bull 513.

172 Doris (n 3) 72–85.

173 AM Isen and PF Levin, ‘Effect of Feeling Good on Helping: Cookies and Kindness’ (1972) 21 J Pers Soc Psychol 384.

174 JM Darley and CD Batson, ‘“From Jerusalem to Jericho": A Study of Situational and Dispositional Variables in Helping Behaviour’ (1973) 27 J Pers Soc Psychol 100.

175 S Milgram, *Obedience to Authority* (Harper and Row 1974).

176 Doris (n 3) 39–51.

177 ibid.

178
*W v M* (n 14).

179
*James* (n 15); *NHS v DJ* (n 78); *NHS v James* (n 78).

180
*Lincolnshire v N* (n 38).

181
*M v N* (n 38).

182
*Montgomery v Lanarkshire* (n 150).

183 J Lothe and J Hawthorn, *Narrative Ethics* (Rodopi 2013).

